# A Potential Oncoprotective Role of Cytomegalovirus Against Breast Cancer: Worldwide Correlation and Survey of Evidence

**DOI:** 10.3390/diseases13060181

**Published:** 2025-06-09

**Authors:** Marko Jankovic, Sofija Glumac, Aleksandra Knezevic, Ana Tomic, Danijela Miljanovic, Jovana Cupic, Ana Banko, Djurdjina Kablar, Ivana Celic, Sara Urosevic, Ivana Lazarevic

**Affiliations:** 1 Department of Virology, Institute of Microbiology and Immunology, 1 Dr Subotica Street, 11000 Belgrade, Serbia; aleksandra.knezevic@med.bg.ac.rs (A.K.); danijela.karalic@med.bg.ac.rs (D.M.); ana.banko@med.bg.ac.rs (A.B.); ivana.lazarevic@med.bg.ac.rs (I.L.); 2 Faculty of Medicine, University of Belgrade, 8 Dr Subotica Street, 11000 Belgrade, Serbia; sofijaglumac09@gmail.com (S.G.); ivanka.celic2309@gmail.com (I.C.); urosevicsaraa@gmail.com (S.U.); 3 Institute of Pathology, 1 Dr Subotica Street, 11000 Belgrade, Serbia; 4 Center for Radiology Imaging, University Clinical Center of Serbia, 2 Pasterova Street, 11000 Belgrade, Serbia; anatomic9977@gmail.com; 5 Clinic for Gynecology and Obstetrics, University Clinical Center of Serbia, 11000 Belgrade, Serbia; cupicjovana@gmail.com; 6 Department for Pathology, Pathohistology and Medical Cytology, University Clinical Centre of Serbia, 11000 Belgrade, Serbia; kablar.djurdjina@gmail.com

**Keywords:** cytomegalovirus, breast cancer, oncoprotection, worldwide

## Abstract

Introduction: While not considered a genuine tumorigenic pathogen, the human cytomegalovirus (CMV) has been associated with a wide assortment of malignancies, including breast cancer (BC). In recent years, increasing evidence has been detailing the potential anti-oncogenic capabilities of CMV. Works in the literature addressing the issue are scarce, and a global approach elucidating the role of CMV in breast cancer is lacking. Aim: We inquired into the association between CMV and BC on a global level and surveyed the related literature. Material and Methods: Virus–tumor interaction was examined by correlating country-specific CMV seroprevalence and the age-standardized BC incidence rates for 73 countries, as provided by the International Agency for Research on Cancer (IARC). Statistical analysis was conducted using Spearman’s correlation, along with univariate and multivariate linear regression analysis. The literature review included works available in the PubMed^®^ database until and including February 2025. Results: The worldwide incidence of BC correlated strongly and inversely with CMV prevalence the world over (*p* < 0.001, Spearman ρ = −0.553). This association was upheld after univariate and multivariate linear regression, extending to other tumors such as skin melanoma and kidney cancer (*p* < 0.001). Conclusions: In this study, we draw attention to a previously unexplored global inverse relationship between the prevalence of CMV and the incidence of BC, which suggests a potential oncoprotective role for this pathogen. Although the association itself does not imply causality, these data provide an intriguing possibility of observing CMV as a tentative factor of protection against this malignancy.

## 1. Introduction

The human cytomegalovirus (CMV; *Cytomegalovirus humanbeta5* or human betaherpesvirus 5 [1]) is a ubiquitous pathogen belonging to the *Orthoherpesviridae* family. The virus carries double-stranded DNA within an icosahedral capsid surrounded by a cell-derived phospholipid bilayer and exhibits a high level of intraspecies diversity [1]. It is a frequent cause of human infection, with seroprevalence ranging between 45% and 95%, depending on the population studied [2,3]. Patterns of exposure differ among countries, depending on various factors such as lifestyle, age, geographical area, and social and economic status. While most often causing benign disease in healthy subjects, CMV is a notorious agent of disease in the immunocompromised. In recent years, mounting evidence supports a compelling correlation between CMV and malignant diseases, implying an oncomodulatory role of the pathogen [4,5,6].

The debate regarding CMV oncogenicity is a polarized one. Although CMV is not considered an oncogenic pathogen, it has been associated with a broad swathe of tumors, such as hematopoietic malignancies, glioblastoma, neuroblastoma, prostate cancer, colon cancer, and salivary gland cancer, as well as breast cancer [6,7,8]. CMV can disrupt cellular pathways and processes, potentially increasing cancer susceptibility by affecting the cell cycle, apoptosis, angiogenesis, cell invasion, and the immune response [9,10]. CMV-induced behavior that may well promote cancer survival is also reflected in its ability to impinge upon the immune system. CMV’s ability to evade immunity may promote neoplastic transformation and metastasis [11,12]. Viral inhibition of the apoptosis of tumor cells by several CMV proteins was also noted, along with impaired p53 function, highlighting the virus’s tumorigenic potential.

A growing body of proof, however, speaks in favor of a protective effect that CMV might impart against various forms of neoplasia [13,14,15]. A possible means by which CMV orchestrates an anti-cancer response is by galvanizing a tumoricidal T-cell response, which precludes the formation of a full-blown malignancy [16,17]. Some of the oncoprotective mechanisms by which CMV may bestow a protective effect on its host include the potential inhibition of the migration of specific breast cancer cells by the virus [18], the potential to redirect the T cell immune response specific to CMV toward tumor cells instead, consequently impacting tumor regression [19], the hypothesized use of the CMV gB protein as a means of tumor suppression [18], and viral reactivation as a relapse reduction factor for a variety of hematological malignancies [20,21,22,23], to name just some of them.

Like with other neoplastic dyscrasia, the connection between CMV and breast cancer is yet to be fully elucidated. Studies investigating the role of CMV in this malignancy do not abound, and the ones that do exist are solely single-center experiences. Currently, there is a lack of comprehensive studies that would span various populations and geographical expanses, thereby giving a global perspective on the subject.

We inquired into the association between CMV and breast neoplasia on a worldwide level and surveyed the published literature. This study draws attention to a hitherto unreported inverse correlation between viral pervasiveness and breast tumor incidences the world over. These intriguing and novel findings further advance the idea of CMV as a potential agent of oncoprevention. Expanding on our prior study in this research domain [13], as well as other recent clinical investigations [14,15], we offer a perspective on the potential role of CMV as a viable factor in averting the initiation of cancer across diverse demographic, geopolitical, and socio-economic contexts.

## 2. Materials and Methods

To explore the potential anti-oncogenic effects of CMV, we examined the association between country-specific age-standardized cancer incidence rates and the corresponding CMV seroprevalences.

The age-adjusted annual incidence rates (per 100,000 individuals) for 6 cancer categories have been recorded across 185 countries for the year 2020, sourced from the Global Cancer Observatory (GLOBOCAN), an online database and project of the International Agency for Research on Cancer (IARC), providing global cancer statistics under the aegis of the World Health Organization (WHO) [24]. These incidences were observed collectively for a total of 34 tumors in both males and females, spanning the entire listed age range (0–85+ years). The list of scrutinized malignancies, as well as their association with CMV pervasiveness, is presented in Table 1 and Table 2.

The prevalence of CMV was represented using country-specific seroprevalence data from 73 countries derived from data collected by Zuhair et al. [2], who systematically reviewed the published literature to assess global CMV IgG antibody prevalence.

For each tumor type or localization, univariate and multivariate linear regression analyses were conducted. In the univariate analysis, CMV was included as an independent predictor, based on both the correlation results and expert judgment. Multivariate analyses were adjusted for confounding variables, which were selected based on their documented association with CMV prevalence: human development index (HDI, which is used as a stand-in for socioeconomic status, or SES); average population age; smoking; breastfeeding; and with HDI being used as a parallel of socioeconomic status, encompassing three major factors: life expectancy, education (mean years of schooling completed and expected years of schooling upon entering the education system), and per capita income.

As regards the literature search, the PubMed^®^ engine was used in order to obtain all relevant references. Studies listed in this manner were further interrogated for relevant publications, i.e., those that pertain to the underlying issue. Furthermore, works referenced within the acquired studies were additionally scoured for pertinent information and included herein. Original papers and reviews in the English language were utilized, along with papers where abstracts were the only available section of the work. All the search was completed in March 2023.

Statistical investigation, i.e., a comparison between age-standardized annual cancer incidence rates and country-specific CMV seroprevalence, was performed by means of Spearman’s rank correlation test, which was complemented by univariate and multivariate regression analyses. The analyses were conducted using SPSS (IBM Corp. v.20.0, Armonk, NY, USA) statistical software, and *p*-values were used to denote corresponding levels of statistical significance.

## 3. Results

An inverse correlation was apparent between the prevalence of CMV and the following tumor categories: “Melanoma (skin), “Kidney”, “All cancers”, “All cancers (excluding non-melanoma skin cancer)“, and “Breast cancer”. Each of the associations demonstrated a high degree of statistical significance (*p* < 0.001).

The strongest rank correlation coefficient was observed for incidences of melanoma of the skin (Table 1). Although declining in number, the rest of the rho numerical values were quite similar, again indicating the similar strength and same direction of the monotonic relationship between CMV pervasiveness and tumor incidence.

**Figure 1:**
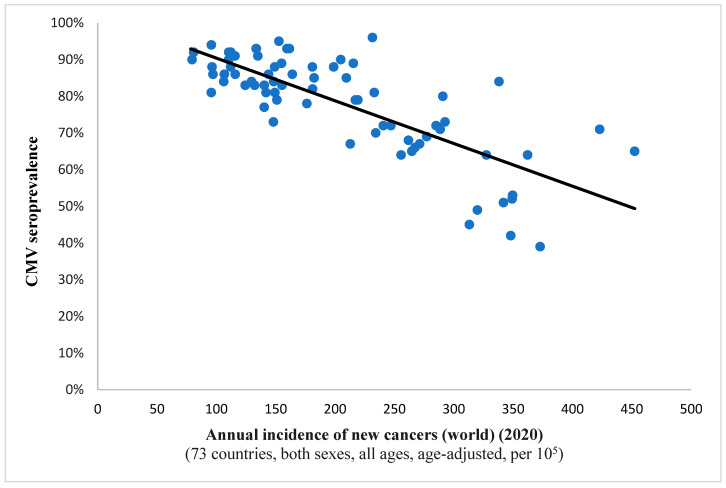
This scatter plot represents a significant relation (Spearman’s ρ = −0.732; *p* < 0.001) between CMV prevalence and the annual incidence of newly detected malignancies the world over for a complement of 73 countries. Namely, as the viral pervasiveness rises, the cancer incidence diminishes, hinting at an antitumor effect of CMV.

**Figure 2:**
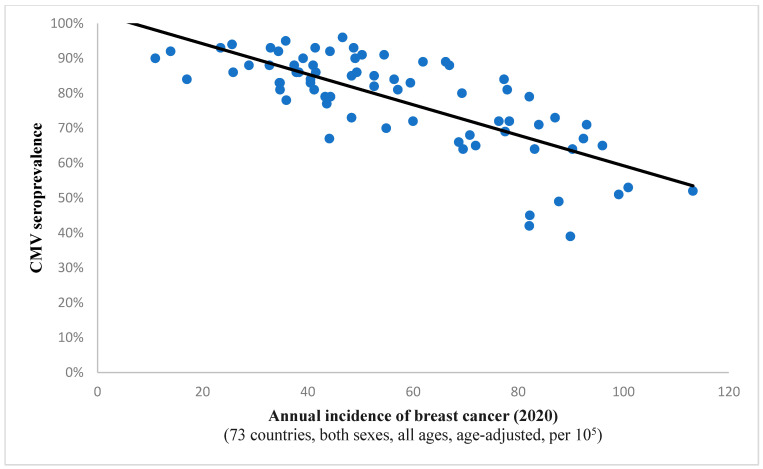
The graph showcases the relationship between breast cancer frequency and CMV prevalence in 73 states across the globe. The strong and inverse correlation (Spearman’s ρ = −0.719; *p* < 0.001) suggests a potential protective effect of the virus against oncogenesis in this case.

It is interesting to note the continued existence of this statistical correlation when examining incidence rates for all cancers combined (Spearman’s ρ = −0.732, *p* < 0.001; depicted in Figure 1). This finding implies a potential protective effect of the virus against all included neoplastic conditions on a global level. A comparable observation was noted in the case of breast cancer, suggesting a potential anti-tumor characteristic of the pathogen within this histological context as well (Figure 2).

No correlation was evident between CMV seroprevalence and the frequency of Kaposi’s sarcoma (Spearman’s ρ = −0.007, *p* = 0.953; shown in Table 1). The mentioned tumor was used in this study as a control variable against which other associations may be compared. This is in accordance with the idea of CMV-stimulated T cell tumoricidal activity [16,17,25]; namely, that individuals affected by HIV/AIDS exhibit marked impairment of the T cell immune response and are particularly affected by the prominent occurrence of Kaposi’s sarcoma.

An analysis of univariate linear regression (ULR) was conducted, with CMV being used as an independent variable. Upon implementing univariate linear regression analysis, the association (previously observed by Spearman’s correlation) still maintained both significance (*p* < 0.001) and direction (Cf. standardized β coefficient) for breast cancer and melanoma, as well as kidney malignancies. Notably, the correlation was also preserved for all tumors worldwide.

The prevalence of CMV is a substantial and independent predictor of tumor incidence. This means that a rise in CMV prevalence is strongly and highly correlated with a reduction in the occurrence of mentioned cancers. When exploring the effect of HDI as a potential confounder in multiple linear regression analysis, CMV remained a strong predictor for the reduced frequency of tested tumors, displaying favorable overall MLR model characteristics. As was expected, the univariate linear regression uncovered a lack of association with Kaposi’s sarcoma (*p* = 0.255). The MLR analysis revealed no significant changes in the results after adjusting for confounding factors such as age, smoking, and breastfeeding. Consequently, these variables were excluded from the final model.

The results of the uni- and multivariate analyses can be found in Table 2 and Table 3.

## 4. Discussion

The interplay between CMV and tumors is the subject of ongoing discussion. Despite numerous instances demonstrating CMV’s anti-tumor properties, the broader public perception appears to focus on its potential for oncomodulation (making malignant tumors even more aggressive) and its suspected pro-oncogenic influence. In this study, we explored the association of CMV seroprevalence with breast cancer, a malignant disorder that stood as the most prevalent cancer at the conclusion of 2020.

### 4.1. Arguments in Favor of CMV-Mediated Carcinogenesis and Oncomodulation

Up until now, the potential association of CMV with malignancies has remained the subject of ongoing discussion. Its potential to induce cancer has been suggested on multiple occasions [26,27], and a number of researchers advocate for the tumorigenic effect of CMV [7,8,28,29,30], with some studies proposing its involvement in more than 90% of commonly occurring tumors [31]. The pathogen has been associated with a wide panoply of malignancies, such as hematopoietic malignancies, glioblastoma, neuroblastoma, prostate cancer, colon cancer, and salivary gland cancer, as well as breast cancer [6,7,8,32]. It is suggested that CMV has the capacity to impact cellular processes and pathways, potentially heightening the vulnerability of cells to cancer development by disrupting the pathways linked to the cell cycle, apoptosis, angiogenesis, cell invasion, and the host’s immune response [9,10].

Studies have identified CMV as a potential risk factor for breast cancer; notably, in individuals with this malignancy, those who tested positive for CMV or had CMV DNA in the tumor tissue were more predisposed to develop stage IV metastatic tumors, implying a role of the virus in promoting metastases [33], while a link between the clinical stage and CMV infection was also observed elsewhere [34]. Additionally, the expression of viral proteins was linked to reduced overall survival in breast cancer patients [35], and the expression of the CMV *IE2* gene was also correlated with this type of tumor [36]. A relationship between CMV infection and breast cancer prognosis has also been noted in a study by Touma et al. [35].

CMV-induced behavior that may well promote cancer survival is also reflected in its impinging on the immune system. In the study by Geisler et al., it was concluded that CMV-infected cells possess the capability to escape recognition and clearance by the immune system through strategic manipulation, impeding antigen presentation by expressing molecules that inhibit T cells and also regulating immune-suppressive type II macrophages [37]. Other investigations are also congruent with the abovementioned studies, suggesting that CMV infection in macrophages promotes a phenotype resembling TAM (tumor-associated macrophages), aiding in neoplastic transformation by evading the immune system, which could potentially lead to metastatic events [11]. Alsamarai and colleagues have demonstrated that CMV IE1, IE2, UL36, UL37, and UL38 proteins effectively inhibit the apoptosis of tumor cells by avoiding immune elimination by NK and cytotoxic T cells [12]. Apparently, cytomegalovirus also has negative effects on the function of p53, which contributes significantly to our comprehension of the virus’s tumorigenic characteristics [38]. Curiously enough, although tested in a cohort of women with benign breast disease and carcinoma, no connection was noted between CMV IgG and diagnosis; the authors note that viral infection influences cytokine production and contributes to changed cytokine profiles in this tumor type [39].

Our current findings suggest that CMV infection is more prevalent in breast carcinomas than in non-tumor tissues. Furthermore, the proposed pathogen-driven breast carcinogenesis model indicates that a number of oncogenic pathways may be activated by viral oncoproteins, promoting cell proliferation, survival, and the development of tumors [40].

At the very least, CMV seems to play a part in oncomodulation [4,41,42,43], supporting cancer proliferation and longevity [5,44] and enhancing its malignant potential by inducing the transition to malignant phenotypes. Haidar Ahmad et al. demonstrated that the virus prompts the heightened expression of specific oncogenes and genes associated with cell survival and cell proliferation (e.g., the *Ki67* gene), as well as DNA reparation and the EMT (epithelial-mesenchymal transition) [45]. Other authors have explored the influence of CMV infection on the development of tumor metastases, suggesting that it facilitates cell invasion through interleukin-induced cellular alterations [46,47]. According to Yang and colleagues, the detection of glycoprotein B in a tumor is associated with the occurrence of metastases and the severity of the clinical condition, which could potentially assist in providing adequate therapy for the given case [33]. There seem to be other ways in which the virus creates a pro-oncogenic cellular environment, such as reduced expression of the *p53* gene, enhancing the phosphorylation of the *Rb* gene, increasing telomerase activity, and more [48,49].

It is worth noting, however, that not all oncomodulatory effects necessarily harm the host. Specifically, there have been observations that CMV may inhibit the migration of specific breast cancer cells [18].

The CMV genome contains numerous proteins that promote cancer cell activity. The HCMV IE1 protein, as reported by Yurochko et al. [50], has been shown to induce NF-kB expression and plays a role in activating cell survival pathways in tumor cells. Additionally, Mitchell et al. [51] found that the pp65 protein was expressed in over 90% of glioblastoma multiforme tissues but was not detected in normal brain tissue, suggesting a potential association with the tumor.

Certain high-risk CMV strains have even been implicated in the active transformation of primary cells [6,37,44,52]. Notably, Herbein and Cobbs noted that CMV not only has the ability to modify epithelial cells but also participates in the transformation of epithelial to mesenchymal (EMT) cells in tumor cells and vice versa [6,52]. Furthermore, it has been suggested that high-risk HCMV strains exhibit a direct oncogenic effect that is characterized by enhanced stemness, epithelial-to-mesenchymal transition, cellular stress, and polyploid giant cancer cells (PGCCs), an effect that is eventually conducive to aggressive cancer phenotypes [53]. It is important to recognize that EMT has been suggested as the primary cause of a loss of cell-to-cell adhesion, disrupted cellular polarity, and reconfiguration of the cytoskeleton [54], thereby playing a crucial role in tumor progression and serving as a key target of interest in anti-cancer therapy [55]. Cytomegalovirus might serve as a causative agent in GBM through ARG2 upregulation [56] and STAT3 signaling [9], which is often used as an early tumor biomarker. The chemokine receptor US28 binds and triggers a proliferative response that can promote tumorigenesis [57]. CMV proteins and DNA have been identified in breast ductal carcinoma in situ, as well as infiltrating ductal carcinoma tissues [58]. Finally, a meta-analysis by Richardson and colleagues [59], along with their clinical data, did not establish a definitive etiological link between CMV and breast cancer. However, it did suggest several possibilities: (a) ‘hit and run’ oncogenesis may give rise to varying results, (b) CMV, EBV, or both may contribute to later stages of breast cancer development, (c) limitations in the molecular tests may have influenced the discoveries, (d) co-infection with multiple viruses might increase the peril of breast cancer, or (e) neither pathogen contributes.

All the evidence mentioned here, when taken together, hints at CMV being involved in oncomodulation and, possibly, in frank tumorigenesis for at least certain histologic types of breast cancer [60], although its role cannot be categorically stated as such at this time.

### 4.2. Arguments in Favor of CMV Oncoprevention

Although the literature has, for the most part, supported a potentially tumorigenic effect of CMV, recent years have seen rising evidence of an oncoprotective faculty that the virus might bestow upon its host [13,18,19].

In our investigation, we have described a significant and inverse correlation between the age-standardized incidence of breast cancer and CMV prevalence across 73 countries worldwide. Intriguingly, the data indicate that breast cancer incidences tend to become significantly lower as CMV pervasiveness grows higher. This association hints at a potential oncoprotective effect that CMV infection may proffer against this malignancy. While it is crucial to emphasize that correlation does not imply causation, this association is supported by various other research findings. While evidence for an anti-tumor effect of CMV in breast cancer was hitherto virtually non-existent, it was noted elsewhere that the virus may inhibit the migration of specific breast cancer cells [18].

This study builds upon our previous research on B-cell cancers, wherein we have also demonstrated the same inverse correlation occurring between these malignant dyscrasia and CMV infection [13]. A similar result was observed in a murine CMV experimental model, wherein the virus had an adverse impact on the advancement of B-cell lymphoma [61].

Çuburu et al. illustrated the potential for redirecting the T cell immune response specific to CMV toward tumor cells, consequently impacting tumor regression [19]. Some other studies have described the immune-mediated oncoprotective effect of the virus using antibodies. These effects have also been observed elsewhere, and those surveys indicate that identifying CMV gB as a TGF-β/Smad signaling inhibitor may be beneficial in developing effective therapeutic strategies using anti-TGF-β agents to promote tumor suppression [18].

There is other evidence that points to an oncopreventive faculty of CMV. The virus has shown an anti-leukemia effect in patients undergoing allogeneic hematopoietic stem cell transplantation (HSCT); in this setting, viral reactivation was linked to a noticeable reduction in the risk of disease relapse [20]. The mitigation of risk for disease relapse by CMV reactivation was also observed in the case of non-Hodgkin lymphomas [21], acute myeloid leukemia [22], and pediatric acute leukemia in the wake of HSCT [23]. Viral reactivation after HSCT was associated with a minor decrease in early relapse risk in a group with myeloproliferative disorders [62]. Moreover, the status of CMV did not exhibit a correlation with the risk of developing most cancers in the recipients of solid organ transplants in another study [63].

Cytomegalovirus seropositivity may be influenced by a number of factors: age [64,65,66], sex [64,67,68], childcare practices [65], varied cultural norms or practices associated with breastfeeding [65], level of education [69], number of sexual partners [69], SES [69,70,71], and current smoking [69], to list only some of them. Ethnic or racial background is also linked to SES [72,73]. Certain elements among these examples could be regarded as confounding variables in this examination. However, smoking can be disregarded as it is a pro-oncogenic practice. Also, upon exploring the role of HDI (used as an alternative for SES) as a confounding variable in multiple linear regression analysis, it was noted that CMV continued to be a robust predictor for a decreased occurrence of the examined malignancies, demonstrating favorable characteristics in the overall MLR model. Recently, we have also demonstrated an inverse correlation between a number of tumors, including lung cancer, and CMV seroprevalence, hinting at the potential oncoprevention that CMV infection might impart against malignancies of the lungs [74]. This should be interpreted cautiously, however, as there are many types of lung cancer, and analysis has not been performed on specific tumors per se. Additionally, correlation should not be interpreted unequivocally as causation.

An interesting and very recent finding emerged in the clinical studies published by Rashid et al. [14] and Nagel et al. [15], where anti-tumor effects that were attributed to viral infection were described in persons with bronchogenic and colorectal carcinoma, respectively. Although not a de facto argument in favor of oncoprotection, it should also be noted that a number of research articles mention a notable lack of CMV DNA in a variety of tumors [75,76].

Finally, other preliminary results would seem to suggest that the potential oncopreventive effect of CMV seems to hold true across a spectrum of races/ethnicities on a global scale (Preprint [77]). Namely, the inverse relationship between cancer incidence and CMV seropositivity, both globally and in the U.S. specifically, suggests that CMV may play a protective role against tumorigenesis. This phenomenon maintained a consistent pattern across various racial and ethnic groups within the United States and was observed across a diverse variety of neoplasia. This preliminary research supports CMV’s potential in anti-cancer vaccinology and highlights its ability to inhibit tumor development across diverse populations and cancer types.

All argumentation in favor of a potential CMV oncopreventive faculty may be seen in Table 4.

## 5. Conclusions

A continuous debate surrounds the precise impact of CMV on tumor tissue, with arguments ranging from direct oncogenesis to oncomodulation and oncoprevention. However, the temporal aspect must also be considered, as there is a possibility that the virus exerts a tumorigenic effect at a specific age while providing protection at another. This aspect remains to be thoroughly investigated. Nevertheless, it is indisputable that a connection between CMV and tumors exists, as demonstrated in many studies. Our investigation, along with a very small number of others, has established a link between the anti-cancer effect and breast malignancies. However, this serological evidence requires validation through comprehensive molecular studies, including CMV detection in tumor tissues, and we are still a considerable distance away from fully understanding the true role of CMV in oncogenesis.

## Figures and Tables

**Table 1:** The WHO’s GLOBOCAN tumor data as regards global CMV prevalence, analyzed with correlation and regression, suggest that CMV may offer oncoprotection, as higher CMV prevalence correlates with lower tumor incidence in significant cases.

Tumor/Localization	Correlation Analysis		Univariate Linear Regression Analysis				Multivariate Linear Regression Analysis **			
	Spearman’s ρ	*p*-Value	Stand. Coeff. β	*p*-Value	R^2^	Adj. R^2^	Stand. Coeff. β	*p*-Value	R^2^	Adj. R^2^
1. Melanoma (skin)	−0.763	0.001 *	−0.719	<0.001 *	0.518	0.511	−0.529	<0.001 *	0.573	0.561
2. Kidney	−0.754	0.001 *	−0.792	<0.001 *	0.627	0.622	−0.493	<0.001 *	0.771	0.765
3. Breast	−0.719	0.001 *	−0.754	<0.001 *	0.569	0.563	−0.470	<0.001 *	0.690	0.681
4. Kaposi’s sarcoma ^†^	−0.007	0.953	0.135	0.255	0.018	0.004	0.031	0.836	0.041	0.013
5. All cancers	−0.732	0.001 *	−0.776	<0.001 *	0.603	0.597	−0.482	<0.001 *	0.745	0.737
6. All cancers (excluding skin non-melanoma)	−0.726	0.001 *	−0.778	<0.001 *	0.605	0.599	−0.462	<0.001 *	0.770	0.763

*Stand. Coeff. Β*—Standardized coefficients β; Adj. R^2^—adjusted R^2^. * Asterisks denote statistically significant correlations; significance is at *p* < 0.05; ** Multivariate regression analysis, adjusted for HDI. ^†^ Kaposi’s sarcoma was selected for the study as a control parameter for CMV-derived T cell tumoricidal activity (Cf. Section 3).

**Table 2:** Univariate linear regression analysis with model characteristics.

	Model Characteristics		Univariate Linear Regression Analysis		
Tumor/Localization	R^2^	Adjusted R^2^	Standardized Coefficients β	95% CI	*p*-Value
1. Melanoma (skin)	0.518	0.511	−0.719	−55.518–−34.870	<0.001
2. Kidney	0.627	0.622	−0.792	−24.206–−16.731	<0.001
3. Breast	0.569	0.563	−0.754	−157.096–−103.411	<0.001
4. Kaposi’s sarcoma	0.018	0.004	0.135	−0.626–2.321	0.255
5. All cancers	0.603	0.597	−0.776	−617.383–−418.339	<0.001
6. All cancers (excluding skin non-melanoma)	0.605	0.599	−0.778	−511.328–−347.189	<0.001

**Table 3:** Multivariate linear regression analysis with model characteristics.

	Multivariate Linear Regression Analysis				Model Characteristics	
Tumor/Localization	Factor	Stdand. Coeff. β	95% Confidence Interval (CI)	*p*-Value	R^2^	Adjusted R^2^
1. Melanoma (skin)	CMV	−0.529	−46.017–−20.828	<0.001	0.573	0.561
	HDI	0.306	6.219–29.515	<0.001		
2. Kidney	CMV	−0.493	−16.622–−9.031	<0.001	0.771	0.765
	HDI	0.484	8.128–15.149	<0.001		
3. Breast	CMV	−0.470	−110.714–−51.989	<0.001	0.690	0.681
	HDI	0.454	45.398–99.712	<0.001		
	HDI	0.244	−1.787–17.442	0.109		
4. Kaposi’s sarcoma	CMV	0.031	−1.695–2.088	0.836	0.041	0.013
	HDI	−0.182	−2.812–0.686	0.230		
	HDI	0.065	−8.125–12.595	0.668		
5. All cancers	CMV	−0.482	−427.382–−220.202	<0.001	0.745	0.737
	HDI	0.478	200.918–392.535	<0.001		
6. All cancers (excluding skin non-melanoma)	CMV	−0.462	−338.43–−175.618	<0.001	0.770	0.763
	HDI	0.513	188.415–338.997	<0.001		

**Table 4:** Observations of the potential CMV oncopreventive effect against various tumors.

Statement/Conclusion	Reference No. (This Work)
Inhibition of tumor cell migration CMV gB as a potential TGF-β/Smad signaling inhibitor	[18]
Adverse impact on the advancement of B-cell lymphoma in a murine model	[61]
Immune-mediated oncoprotective effect	[19]
Mitigation of risk for disease relapse	[20,21,22,23,62]
Lack of connection between CMV serology and a number of solid organ tumors	[63]
Inverse correlation between CMV seroprevalence and tumor frequency	[13,74,77]
Lack of CMV DNA in a variety of tumors	[75,76]
Prior CMV infection reduces tumor incidence	[14,15]

## Data Availability

The original contributions presented in this study are included in the article. Further inquiries can be directed to the corresponding author.
